# Association of coexisting type 2 diabetes and DXA-defined low muscle mass with prevalent radiographic vertebral fracture in community-dwelling older adults: a cross-sectional study

**DOI:** 10.3389/fpubh.2026.1908757

**Published:** 2026-09-16

**Authors:** Xuanpu Zhong, Hailong Wu, Tianjiao Gao, Zhenwei Zhou, Luan Li, Ling Hao, Yitong Xu, Pengcheng Li, Haisi Dong, Zongjian Luo

**Affiliations:** 1 School of Traditional Chinese Medicine, Changchun University of Chinese Medicine, Changchun, China; 2 Department of Orthopedics, The Affiliated Hospital of Changchun University of Chinese Medicine, Changchun, China; 3 The Affiliated Hospital of Changchun University of Chinese Medicine, Changchun, China; 4 Northeast Asia Research Institute of Traditional Chinese Medicine, Changchun University of Chinese Medicine, Changchun, China; 5 Rehabilitation Teaching and Research Section, Changchun Medical College, Changchun, China

**Keywords:** community-dwelling older adults, cross-sectional study, DXA-defined low muscle mass, healthy aging, prevalence ratio, radiographic vertebral fracture, type 2 diabetes mellitus

## Abstract

**Background:**

Prevalent radiographic vertebral fracture is frequently clinically unrecognized in older adults, and areal bone mineral density does not fully characterize its distribution. Whether coexisting type 2 diabetes mellitus (T2DM) and objectively measured low muscle mass identify a subgroup in which this burden is concentrated remains unclear.

**Methods:**

This community-based cross-sectional analysis included 1,642 adults aged 65 years or older recruited through 16 community health service centers covering 36 administrative communities in Changchun, China, between July 2021 and March 2023. Participants were classified into four mutually exclusive groups: neither condition, T2DM alone, dual-energy X-ray absorptiometry (DXA)-defined low muscle mass alone, or both conditions. The primary outcome was prevalent radiographic vertebral fracture (Genant grade ≥ 1), with Genant grade ≥ 2 fracture as the key secondary outcome. Modified Poisson models estimated prevalence ratios (PRs). The primary model included age and body mass index as restricted cubic splines, sex, and current smoking, with covariance estimation accounting for 36 community clusters. Missing covariates were handled using 50 datasets generated by multiple imputation by chained equations, and marginal standardization was used to estimate prevalence differences.

**Results:**

The analytic cohort included 343 participants (20.9%) with prevalent radiographic vertebral fracture. The coexisting phenotype comprised 14.1% of the cohort but accounted for 31.5% of all Genant grade ≥ 1 events and 35.2% of all Genant grade ≥ 2 events. Observed prevalence was 11.5, 20.7, 24.2, and 46.6% in the neither-condition, T2DM-alone, low-muscle-mass-alone, and coexisting groups, respectively. Primary-model PRs versus neither condition were 1.66 (95% CI, 1.30–2.12), 1.92 (95% CI, 1.42–2.59), and 3.63 (95% CI, 2.81–4.68), respectively. The coexisting phenotype had a standardized prevalence of 44.1% (95% CI, 38.3–49.9%) and a prevalence difference of 31.9 percentage points (95% CI, 25.3–38.5) versus neither condition. Results were similar in the femoral-neck-BMD-context, complete-case, Genant grade ≥ 2, and no-prior-fracture analyses.

**Conclusion:**

In this analytic sample, coexisting T2DM and DXA-defined low muscle mass defined a relatively small subgroup with a disproportionate share of prevalent radiographic vertebral fracture events and the highest fracture prevalence. These cross-sectional findings do not establish causality, future fracture prediction, screening performance, or an indication for vertebral imaging.

## 1 Introduction

Prevalent radiographic vertebral fracture is common in older adults and is often detected on imaging without a corresponding clinical fracture history (1, 2). It is associated with subsequent skeletal and functional vulnerability (3, 4), yet many affected individuals remain clinically unrecognized (1, 2). Because the semiquantitative Genant method identifies radiographic vertebral fracture or deformity rather than its etiology, we use the neutral term prevalent radiographic vertebral fracture throughout (5).

Areal bone mineral density (BMD) alone does not fully characterize the distribution of radiographic vertebral fracture (6, 7). In older adults, existing vertebral fracture burden may also reflect diabetes-related impairment in bone quality and turnover (7–9), fall susceptibility and impaired muscle health (10–12), and additional vulnerability related to chronic kidney disease, multimorbidity, and frailty (13–15). Examining these conditions jointly may therefore show whether prevalent events are concentrated in a clinically recognizable subgroup beyond what is apparent from isolated skeletal measures. Such a description remains distinct from causal or predictive inference.

Type 2 diabetes mellitus (T2DM) is particularly relevant because skeletal fragility in diabetes is not fully explained by low areal BMD; people with T2DM may have preserved or higher BMD while remaining susceptible to fracture (6–9, 16). Muscle health adds a related but distinct dimension. Low muscle mass, low strength, and slow physical performance are separate components of the sarcopenia construct and are associated with falls, fractures, mobility limitation, and frailty-related vulnerability (10–12, 17–21). Accordingly, the primary muscle exposure in this study is DXA-defined low muscle mass; clinical sarcopenia also requires an appropriate functional component under the Asian Working Group for Sarcopenia (AWGS) 2019 algorithm (22).

Most prior work has examined diabetes, muscle impairment, osteoporosis, or vertebral fracture separately (6, 17, 23, 24). It remains unclear whether older adults with both T2DM and objectively measured low muscle mass have a disproportionate burden of prevalent radiographic vertebral fracture. For a common cross-sectional outcome, prevalence ratios (PRs), standardized prevalence, and prevalence differences provide complementary relative and absolute descriptions (25, 26). We therefore examined phenotype distribution, event concentration, observed and standardized prevalence, prevalence differences, and PRs across four mutually exclusive T2DM-low-muscle-mass phenotypes. The objective was descriptive and associational, not to establish causal mechanisms or temporal ordering, predict future fracture, assess diagnostic or screening performance, or recommend vertebral imaging.

## 2 Materials and methods

### 2.1 Study design, setting and participants

We conducted a community-based cross-sectional analysis of baseline data collected in Changchun, Northeast China, between July 2021 and March 2023. Nineteen candidate community health service centers were considered; 16 initiated recruitment and covered 36 administrative communities in nine study areas, whereas three did not initiate recruitment and contributed no resident-level records. Sites were selected through a structured, nonprobability process based on geographic coverage, the local older population, availability of community health records, organizational capacity, and feasibility of the standardized baseline assessment. Selection was unrelated to T2DM, low muscle mass, osteoporosis, previous fracture, or vertebral imaging findings, and no disease quotas or phenotype-targeted recruitment were used.

Within the participating service areas, candidate resident records were assembled from several nonspecialty community sources, including community health records, lists of residents aged 65 years or older, older-adult health-examination lists, family-physician enrollment lists, and supplementary community registers. Records were merged and deduplicated before eligibility screening and before classification of T2DM, low muscle mass, or vertebral imaging outcomes. Community staff and family physicians invited residents through telephone or text contact, community messaging channels, and community notices. Eligibility for the present analysis required age ≥ 65 years, residence eligibility within a participating service area, written informed consent, completion of the site baseline assessment workflow, and ascertainable T2DM status, DXA-defined low-muscle-mass status, and baseline vertebral imaging outcome. Supplementary Figure S1 shows the complete participant flow and mutually exclusive first-exit counts.

The analytic sample therefore comprised all recruited participants who met these eligibility and ascertainability requirements; it was not selected through a *post hoc* power calculation. The study protocol was reviewed and approved by the Ethics Committee of the Affiliated Hospital of Changchun University of Chinese Medicine (approval No. CCZYFYLL2021-033). All participants provided written informed consent before baseline assessment, and data were de-identified before analysis.

### 2.2 Ascertainment of T2DM

T2DM status was ascertained from community health-service records and corroborated at the baseline interview by the participant or, when necessary, a family member. Participants were classified as having T2DM when community records documented a clinical diagnosis or the participant or family reported a physician diagnosis. Glucose-lowering medication use was recorded as supporting clinical information but did not determine classification by itself. Baseline glycated hemoglobin (HbA1c) characterized contemporaneous glycemic status but was not used to diagnose or reclassify T2DM. The ascertainment framework was based on existing clinical diagnosis rather than cohort-wide fasting-glucose screening or retrospective biochemical reclassification. Among participants with T2DM, diabetes duration was obtained from medical records when available and otherwise from reported time since diagnosis.

### 2.3 DXA assessment, low muscle mass and phenotype classification

Dual-energy X-ray absorptiometry (DXA) examinations were performed with a Hologic Discovery Wi densitometer (Hologic Inc., Marlborough, MA, USA) using the study’s standardized scanning protocol and current quality-control procedures (27). Daily instrument quality control was performed with the manufacturer-supplied spine phantom and quality-control program before participant scanning; scanning was suspended when results fell outside the manufacturer-specified acceptable range. Whole-body DXA provided appendicular skeletal muscle mass, and skeletal muscle index (SMI) was calculated as appendicular skeletal muscle mass divided by height squared (kg/m^2^).

The primary muscle exposure was DXA-defined low muscle mass rather than clinical sarcopenia. Using the Asian Working Group for Sarcopenia (AWGS) 2019 thresholds, low muscle mass was defined with strict less-than cutoffs of SMI < 7.0 kg/m^2^ in men and < 5.4 kg/m^2^ in women (22). Participants were assigned to four mutually exclusive phenotypes: neither T2DM nor low muscle mass, T2DM alone, low muscle mass alone, and coexisting T2DM and low muscle mass.

Handgrip strength and gait speed were used for descriptive characterization and to cross-validate the full AWGS 2019 classification. Low handgrip strength was defined as < 28 kg in men and < 18 kg in women, and slow gait as < 1.0 m/s. Full AWGS classification required low muscle mass plus low handgrip strength or slow gait; severe AWGS classification required all three components (22). Supplementary Table S1 reports this cross-validation; it did not replace or relabel the primary low-muscle-mass exposure, and no duplicate association model was fitted.

### 2.4 Radiographic vertebral fracture assessment

Prevalent radiographic vertebral fracture was assessed on baseline lateral thoracic and lumbar spine images using the semiquantitative Genant method (5). The primary outcome was at least one vertebra with a highest Genant grade ≥ 1. Genant grade ≥ 2 radiographic vertebral fracture was the key secondary outcome and served as a severity-oriented robustness assessment. These classifications identify radiographic fracture or deformity but do not establish an osteoporotic or fragility etiology (5).

Images were reviewed by trained assessors experienced in musculoskeletal imaging according to the study protocol. Discrepant or uncertain readings were resolved by review with a senior assessor. Inter-reader agreement was not quantified for this analysis. Imaging quality control relied on trained assessment and senior review of discrepant or uncertain readings.

### 2.5 Covariates and other baseline measurements

The primary model adjusted for age, sex, body mass index (BMI), and current smoking. Age was obtained from the baseline record; sex was obtained from the record or interview; BMI was calculated as measured weight divided by measured height squared; and current smoking was coded as a binary yes/no variable from the standardized baseline questionnaire. Age and BMI were modeled continuously using restricted cubic splines (28). Femoral-neck bone mineral density (BMD), measured from the Hologic hip scan, was evaluated only in a separate BMD-context model and was not treated as a mediator or as evidence that the association was independent of BMD. CCI and mFI-5 were recorded as total scores rather than component-specific variables. Because standard formulations may incorporate diabetes, neither total was entered into the final models, thereby avoiding possible partial adjustment for the T2DM exposure; no *post hoc* modified score was constructed.

Previous low-trauma fracture, fall in the previous year, low physical activity, lumbar-spine BMD, SMI, handgrip strength, gait speed, HbA1c, procollagen type I N-terminal propeptide (P1NP), beta C-terminal telopeptide of type I collagen (beta-CTX), 25-hydroxyvitamin D [25(OH)D], and estimated glomerular filtration rate (eGFR) were used for descriptive characterization and, where applicable, as auxiliary variables in the imputation model. Previous low-trauma fracture also defined the sensitivity restriction to participants observed without such a history. The standardized baseline variables used in this analysis did not include calcium, phosphate, parathyroid hormone, alkaline phosphatase, detailed previous or current anti-osteoporosis treatment, calcium or vitamin D supplementation, or other bone-active therapies. These factors were not represented by proxy variables. Supplementary Table S2 details the source, assessment, coding, analytic role, and measurement scope for each variable.

### 2.6 Missing data

Age, sex, T2DM status, SMI, DXA-defined low muscle mass, handgrip strength, gait speed, HbA1c, and both vertebral imaging outcomes were complete in the analytic cohort. Missingness in the remaining variables was low, ranging from 0.79% for current smoking to 3.11% for P1NP. T2DM duration was structurally not applicable to participants without T2DM and was not treated as missing. Variable-specific completeness and missingness are reported in Supplementary Table S3.

The primary and BMD-context analyses used multiple imputation by chained equations for missing covariates (29). Fifty independent chains were run for 30 cycles, and one completed dataset was retained from each chain. Predictive mean matching with five donors was used for continuous variables and logistic imputation for binary variables. The random seed was 20260820. Exposure variables, vertebral fracture outcomes, and eligibility for the no-prior-fracture restriction were not imputed. Estimates were combined across the 50 datasets using Rubin and Barnard-Rubin rules. The complete-case sensitivity analysis included participants with complete data for the primary-model covariates.

### 2.7 Statistical analysis

Continuous variables were summarized as mean (standard deviation) or median (interquartile range), as appropriate, and categorical variables as *n* (%) using variable-specific available-case denominators. Baseline characteristics were described without group-comparison *p* values. Phenotype-specific observed prevalence and Wilson 95% confidence intervals (CIs) were calculated for Genant grade ≥ 1 and grade ≥ 2 radiographic vertebral fracture. Cohort share and event share were treated as descriptive measures of concentration rather than attributable fractions.

The primary relative measure was the prevalence ratio (PR). Because the primary outcome was common, PRs and 95% CIs were estimated using modified Poisson regression with robust variance estimation (25, 30). Participants with neither T2DM nor low muscle mass served as the reference. The primary model included the four-category phenotype, age, sex, BMI, and current smoking. Age used a three-knot restricted cubic spline at 66.91, 73.00, and 81.49 years; BMI used knots at 20.8, 25.4, and 31.2 kg/m^2^. The BMD-context model added femoral-neck BMD as a three-knot restricted cubic spline with knots at 0.521, 0.683, and 0.835 g/cm^2^. No probability weights were applied because the nonprobability site-selection design did not assign individual sampling probabilities.

Covariance estimation accounted for the 36 communities using a cluster-robust sandwich estimator with the CR1 finite-cluster correction; inference used 35 cluster degrees of freedom (31). Primary-model standardized prevalence was estimated by predicting each phenotype for every participant over the common covariate distribution and averaging the predictions (26). Prevalence differences were calculated from these marginal standardized estimates, with delta-method variance within each completed dataset and Rubin pooling across datasets. Direct contrasts compared the coexisting phenotype with T2DM alone and with low muscle mass alone on both PR and prevalence-difference scales.

Robustness was assessed in a separate femoral-neck-BMD-context model, using Genant grade ≥ 2 radiographic vertebral fracture as the key secondary outcome, in complete cases, and after restricting the sample to participants observed without previous low-trauma fracture. All tests were two-sided, and nominal *p* values are reported as *p* < 0.001 or to three decimal places. Analyses were performed in Python 3.13.5 using NumPy 2.3.5, SciPy 1.17.0, statsmodels 0.14.6, and scikit-learn 1.8.0. Estimates were interpreted as cross-sectional prevalence associations and concentration of existing radiographic vertebral fracture events, not as causal effects, future risk, diagnostic performance, or screening efficiency.

## 3 Results

### 3.1 Participant flow and baseline characteristics

The source frame contained 2,586 candidate resident records. Before contact, 203 records were excluded because age was < 65 years (*n* = 82), residence eligibility was not met (*n* = 76), or the record belonged to a prior-wave participant (*n* = 45). Of 2,383 residents who passed prescreening, 156 were not successfully contacted. Among 2,227 residents successfully contacted and formally invited, 243 did not accept the invitation (181 declined and 62 provided no final response). Of 1,984 residents who accepted the invitation and scheduled an assessment appointment, 134 did not attend. Fourteen of the 1,850 residents who arrived at the assessment site did not proceed to written consent. Among 1,836 participants who provided written consent, 127 did not complete the site baseline assessment workflow. A further 67 of the 1,709 participants who completed that workflow did not have ascertainable T2DM, DXA-defined low-muscle-mass, or baseline radiographic vertebral fracture status, yielding the final analytic cohort of 1,642 participants (Supplementary Figure S1).

The final cohort had a mean age of 73.8 years and included 895 women (54.5%) (Table 1). It contained 343 participants (20.9%) with prevalent radiographic vertebral fracture (Table 2). The four mutually exclusive phenotypes comprised 710 participants (43.2%) with neither condition, 460 (28.0%) with T2DM alone, 240 (14.6%) with low muscle mass alone, and 232 (14.1%) with both conditions (Table 1; Figure 1A).

**Table 1:** Baseline characteristics of the analytic cohort according to T2DM-low-muscle-mass phenotype.

Characteristic	Overall (*N* = 1,642)	Neither condition (*N* = 710)	T2DM alone (*N* = 460)	Low muscle mass alone (*N* = 240)	Coexisting T2DM + low muscle mass (*N* = 232)
Demographic and lifestyle characteristics					
Age, years	73.8 (5.5)	72.2 (4.7)	74.3 (5.5)	75.9 (5.6)	75.8 (6.1)
Female sex, *n* (%)	895 (54.5)	367 (51.7)	255 (55.4)	139 (57.9)	134 (57.8)
BMI, kg/m^2^	25.7 (4.0)	24.9 (3.4)	27.4 (4.5)	24.0 (2.9)	26.6 (4.3)
Current smoking, *n* (%)	334 (20.5)	124 (17.7)	108 (23.6)	41 (17.2)	61 (26.3)
Previous low-trauma fracture, *n* (%)	146 (9.0)	44 (6.2)	40 (8.8)	28 (11.9)	34 (14.7)
Fall in previous year, *n* (%)	326 (20.1)	114 (16.3)	104 (22.8)	60 (25.4)	48 (20.9)
Low physical activity, *n* (%)	629 (39.0)	217 (31.0)	178 (39.4)	105 (45.1)	129 (57.3)
Diabetes-related characteristics					
HbA1c, %	6.4 (1.0)	5.7 (0.3)	7.2 (0.8)	5.8 (0.3)	7.6 (0.9)
T2DM duration among participants with T2DM, years	9.0 (6.3)	NA	8.5 (6.1)	NA	9.9 (6.6)
Skeletal and body-composition characteristics					
Femoral-neck BMD, g/cm^2^	0.680 (0.119)	0.704 (0.107)	0.685 (0.119)	0.647 (0.127)	0.626 (0.122)
Lumbar-spine BMD, g/cm^2^	0.880 (0.147)	0.903 (0.138)	0.872 (0.151)	0.863 (0.154)	0.841 (0.145)
SMI, kg/m^2^	6.47 (0.97)	6.95 (0.72)	6.80 (0.69)	5.47 (0.70)	5.38 (0.72)
Handgrip strength, kg	24.7 (7.6)	27.9 (7.2)	26.3 (6.9)	18.6 (4.6)	18.1 (4.4)
Gait speed, m/s	0.95 (0.24)	1.07 (0.18)	0.99 (0.20)	0.74 (0.18)	0.70 (0.16)
Biochemical and renal characteristics					
P1NP, ng/mL	42.0 (32.5–55.7)	37.7 (30.6–47.9)	41.4 (32.1–55.0)	50.5 (38.8–65.1)	52.6 (40.8–73.1)
beta-CTX, ng/mL	0.301 (0.199–0.450)	0.253 (0.168–0.378)	0.297 (0.206–0.417)	0.363 (0.244–0.554)	0.419 (0.290–0.609)
25(OH)D, ng/mL	23.0 (7.9)	25.3 (7.4)	22.7 (8.1)	19.8 (7.4)	19.9 (7.3)
eGFR, mL/min/1.73 m^2^	76.3 (15.9)	81.2 (14.5)	73.2 (15.9)	74.5 (15.8)	69.5 (15.7)

Values are mean (standard deviation), median (interquartile range), or n (%), using variable-specific available-case denominators. Percentages for categorical characteristics use the nonmissing denominator within each phenotype. T2DM duration is summarized only among participants with T2DM and is not applicable to the other phenotype groups. No group-comparison *p* values are presented because the table is descriptive and *p* values were not used to select the adjustment set. BMD, bone mineral density; BMI, body mass index; beta-CTX, beta C-terminal telopeptide of type I collagen; eGFR, estimated glomerular filtration rate; HbA1c, glycated hemoglobin; NA, not applicable; P1NP, procollagen type I N-terminal propeptide; SMI, skeletal muscle index; T2DM, type 2 diabetes mellitus; 25(OH)D, 25-hydroxyvitamin D.

**Table 2:** Prevalent radiographic vertebral-fracture burden and primary-model estimates across T2DM-low-muscle-mass phenotypes.

Phenotype	*n* (%)	Prevalent radiographic VF, *n*; % (95% CI)	Share of all VF events, %	Genant grade ≥ 2 VF, *n*; % (95% CI)	Share of all Genant grade ≥ 2 events, %	Primary-model PR (95% CI); *p*	Standardized prevalence, % (95% CI)	Prevalence difference vs neither, percentage points (95% CI)
Neither	710 (43.2)	82; 11.5% (9.4–14.1%)	23.9	37; 5.2% (3.8–7.1%)	20.3	Reference	12.1% (9.7–14.6%)	Reference
T2DM alone	460 (28.0)	95; 20.7% (17.2–24.6%)	27.7	48; 10.4% (8.0–13.6%)	26.4	1.66 (1.30–2.12); *p* < 0.001	20.1% (16.4–23.9%)	8.0 (3.9–12.0)
Low muscle mass alone	240 (14.6)	58; 24.2% (19.2–30.0%)	16.9	33; 13.8% (10.0–18.7%)	18.1	1.92 (1.42–2.59); *p* < 0.001	23.3% (18.9–27.6%)	11.1 (5.7–16.5)
Coexisting T2DM + low muscle mass	232 (14.1)	108; 46.6% (40.2–53.0%)	31.5	64; 27.6% (22.2–33.7%)	35.2	3.63 (2.81–4.68); *p* < 0.001	44.1% (38.3–49.9%)	31.9 (25.3–38.5)

Observed prevalence confidence intervals are Wilson 95% confidence intervals. Primary-model PRs, standardized prevalence, and prevalence differences were derived from the modified Poisson model with age and BMI modeled using three-knot restricted cubic splines, sex, and current smoking. Fifty multiple-imputation completed datasets were pooled using Rubin rules, and covariance estimation accounted for 36 community clusters with the CR1 small-sample correction. Neither condition is the reference phenotype. Prevalence differences were calculated from the unrounded pooled marginal standardized prevalence estimates; therefore, they may not equal the arithmetic difference between the displayed one-decimal prevalences. Event shares are descriptive concentration measures and are not attributable fractions. CI, confidence interval; PR, prevalence ratio; T2DM, type 2 diabetes mellitus; VF, vertebral fracture.

**Figure 1:**
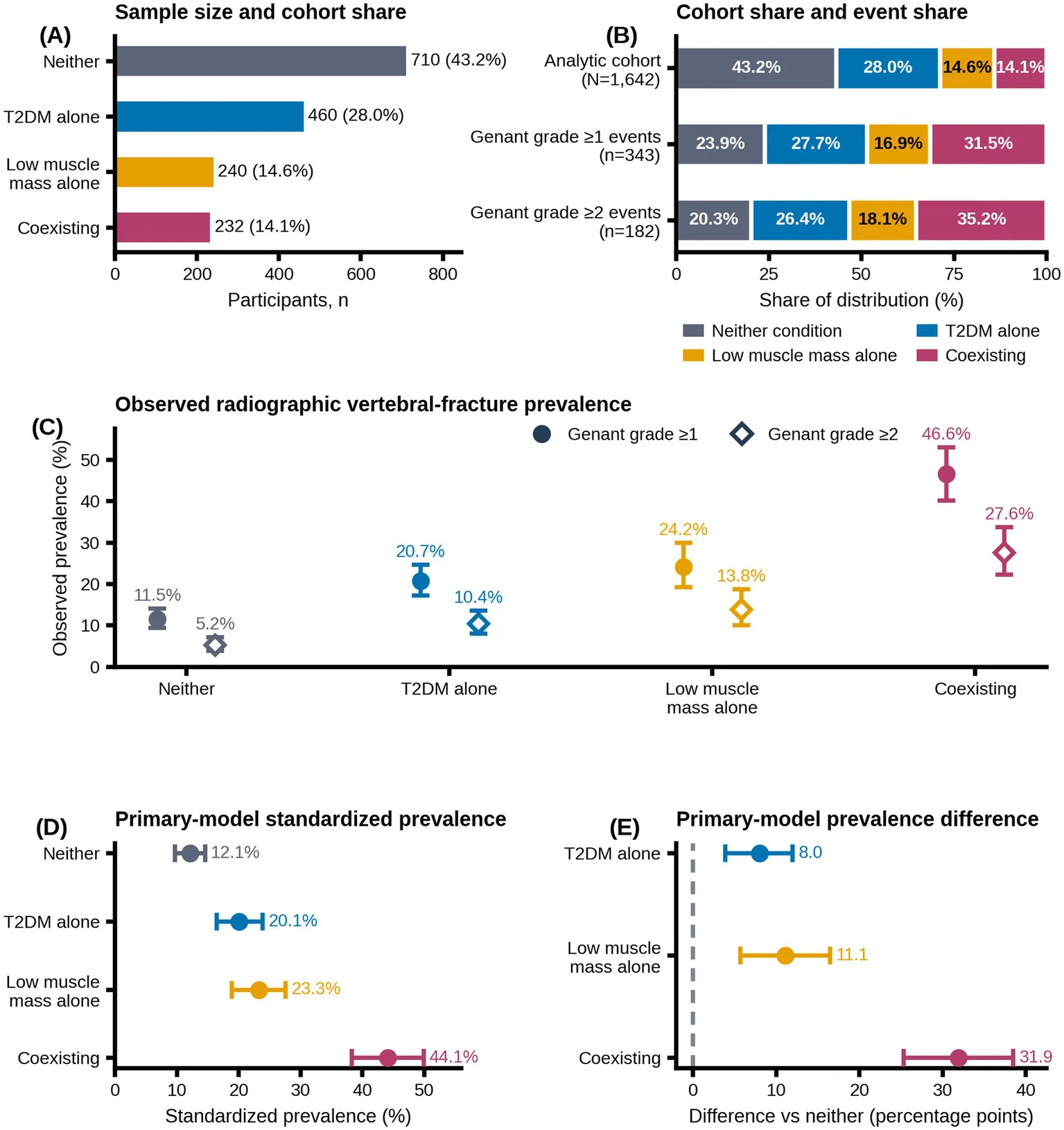
Absolute burden of prevalent and Genant grade ≥ 2 radiographic vertebral fracture across T2DM-low-muscle-mass phenotypes. **(A)** Phenotype-specific sample size and cohort share in the final analytic sample. **(B)** Phenotype composition of the analytic cohort (*N* = 1,642), all Genant grade ≥ 1 radiographic vertebral-fracture events (*n* = 343), and all Genant grade ≥ 2 radiographic vertebral-fracture events (*n* = 182), displayed as shares summing to 100% within each distribution. **(C)** Observed phenotype-specific prevalence of Genant grade ≥ 1 and Genant grade ≥ 2 radiographic vertebral fracture with Wilson 95% confidence intervals. In panel C, colors identify phenotypes and marker shape identifies outcome definition. **(D)** Primary-model standardized prevalence of Genant grade ≥ 1 radiographic vertebral fracture with 95% confidence intervals. **(E)** Primary-model prevalence differences with 95% confidence intervals versus the neither-condition phenotype. Event shares are descriptive concentration measures and represent the distribution of observed events across phenotypes; they are neither within-phenotype prevalences nor attributable fractions. Percentages may not total exactly 100% because of rounding. CI, confidence interval; T2DM, type 2 diabetes mellitus.

Compared descriptively with the neither-condition group, the coexisting group was older and had a higher proportion with low physical activity. It also had lower femoral-neck and lumbar-spine BMD, lower SMI, lower handgrip strength, slower gait speed, higher HbA1c, higher P1NP and beta-CTX, and lower 25(OH)D and eGFR. Among participants with T2DM, mean diabetes duration was 9.9 years in the coexisting group and 8.5 years in the T2DM-alone group. These characteristics are reported with variable-specific available-case denominators in Table 1.

All 472 participants classified with DXA-defined low muscle mass also met the full AWGS 2019 classification in this analytic sample because each also had low handgrip strength or slow gait; 365 met the severe AWGS classification. This numerical equivalence was used only for cross-validation and did not alter the primary exposure label or model (Supplementary Table S1).

### 3.2 Observed event concentration and absolute prevalence

Observed prevalence of Genant grade ≥ 1 radiographic vertebral fracture was 11.5% with neither condition, 20.7% with T2DM alone, 24.2% with low muscle mass alone, and 46.6% with both conditions. The corresponding prevalence of Genant grade ≥ 2 fracture was 5.2, 10.4, 13.8, and 27.6%, respectively (Table 2; Figure 1C).

The coexisting phenotype represented 14.1% of the cohort but contained 31.5% of all Genant grade ≥ 1 events and 35.2% of all Genant grade ≥ 2 events. By comparison, the neither-condition phenotype accounted for 43.2% of the cohort and 23.9 and 20.3% of the two event sets, respectively (Table 2; Figures 1A,B).

Under the primary model, standardized prevalence was 12.1% (95% CI, 9.7–14.6%) for neither condition, 20.1% (95% CI, 16.4–23.9%) for T2DM alone, 23.3% (95% CI, 18.9–27.6%) for low muscle mass alone, and 44.1% (95% CI, 38.3–49.9%) for the coexisting phenotype (Table 2; Figure 1D).

Compared with neither condition, the prevalence differences were 8.0 percentage points (95% CI, 3.9–12.0) for T2DM alone, 11.1 percentage points (95% CI, 5.7–16.5) for low muscle mass alone, and 31.9 percentage points (95% CI, 25.3–38.5) for the coexisting phenotype (Table 2; Figure 1E).

### 3.3 Primary relative and direct phenotype contrasts

Unadjusted modified Poisson PRs versus neither condition were 1.79 (95% CI, 1.41–2.26) for T2DM alone, 2.09 (95% CI, 1.56–2.81) for low muscle mass alone, and 4.03 (95% CI, 3.11–5.22) for the coexisting phenotype (Figure 2A; Supplementary Table S4A).

**Figure 2:**
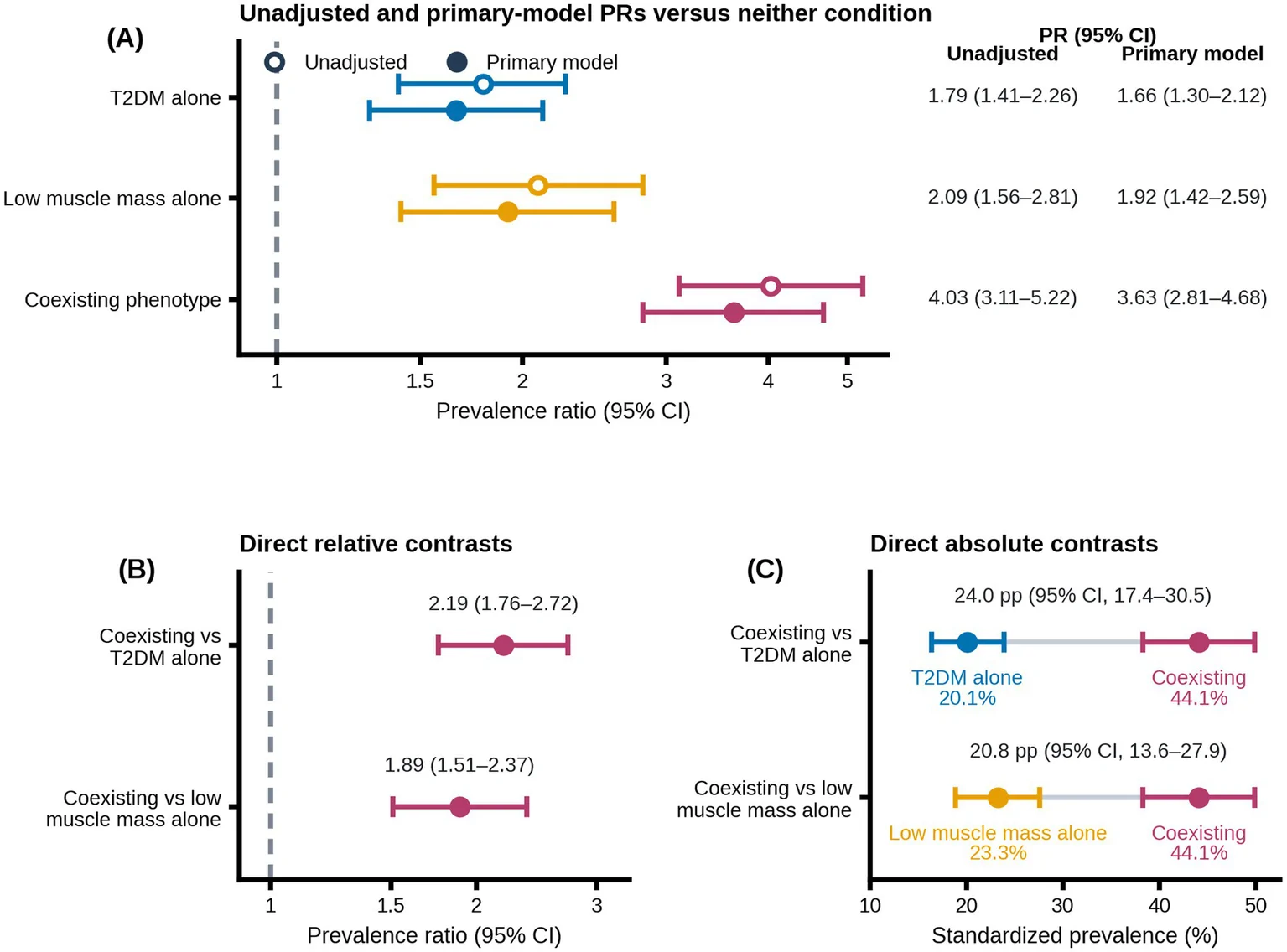
Primary relative and direct absolute contrasts for prevalent radiographic vertebral fracture across T2DM-low-muscle-mass phenotypes. **(A)** Unadjusted and primary-model prevalence ratios for T2DM alone, low muscle mass alone, and the coexisting phenotype versus neither condition. Open points indicate unadjusted estimates and filled points indicate primary-model estimates. No formal test of change between models was performed. **(B)** Direct primary-model prevalence-ratio contrasts comparing the coexisting phenotype with T2DM alone and with low muscle mass alone. **(C)** Primary-model standardized prevalence for the coexisting and single-condition phenotypes, with direct prevalence differences and 95% confidence intervals for the coexisting phenotype versus each single-condition phenotype. The prevalence-ratio axes in panels A and B are shown on a logarithmic scale. Estimates are descriptive cross-sectional contrasts. CI, confidence interval; PR, prevalence ratio; pp., percentage points; T2DM, type 2 diabetes mellitus.

In the primary model, the corresponding PRs were 1.66 (95% CI, 1.30–2.12), 1.92 (95% CI, 1.42–2.59), and 3.63 (95% CI, 2.81–4.68); all *p* < 0.001 (Table 2; Figure 2A; Supplementary Table S4A). Direct comparisons for the coexisting phenotype yielded a PR of 2.19 (95% CI, 1.76–2.72) versus T2DM alone and 1.89 (95% CI, 1.51–2.37) versus low muscle mass alone (Figure 2B; Supplementary Table S4A).

On the absolute scale, standardized prevalence was 44.1% in the coexisting phenotype, compared with 20.1% in T2DM alone and 23.3% in low muscle mass alone. The corresponding direct prevalence differences were 24.0 percentage points (95% CI, 17.4–30.5) and 20.8 percentage points (95% CI, 13.6–27.9), respectively; both *p* < 0.001 (Figure 2C; Supplementary Tables S4B,C).

### 3.4 BMD context, key secondary outcome, and sensitivity analyses

In the femoral-neck-BMD-context model, PRs versus neither condition were 1.64 (95% CI, 1.28–2.11) for T2DM alone, 1.84 (95% CI, 1.36–2.49) for low muscle mass alone, and 3.43 (95% CI, 2.66–4.44) for the coexisting phenotype. Direct PRs for the coexisting phenotype were 2.09 (95% CI, 1.69–2.58) versus T2DM alone and 1.86 (95% CI, 1.50–2.32) versus low muscle mass alone (Figure 3A; Supplementary Table S4A). The standardized prevalence for the coexisting phenotype was 42.4% (95% CI, 36.8–48.0%), with a 30.1-percentage-point difference (95% CI, 23.6–36.5) versus neither condition (Figure 3C; Supplementary Tables S4B,C).

**Figure 3:**
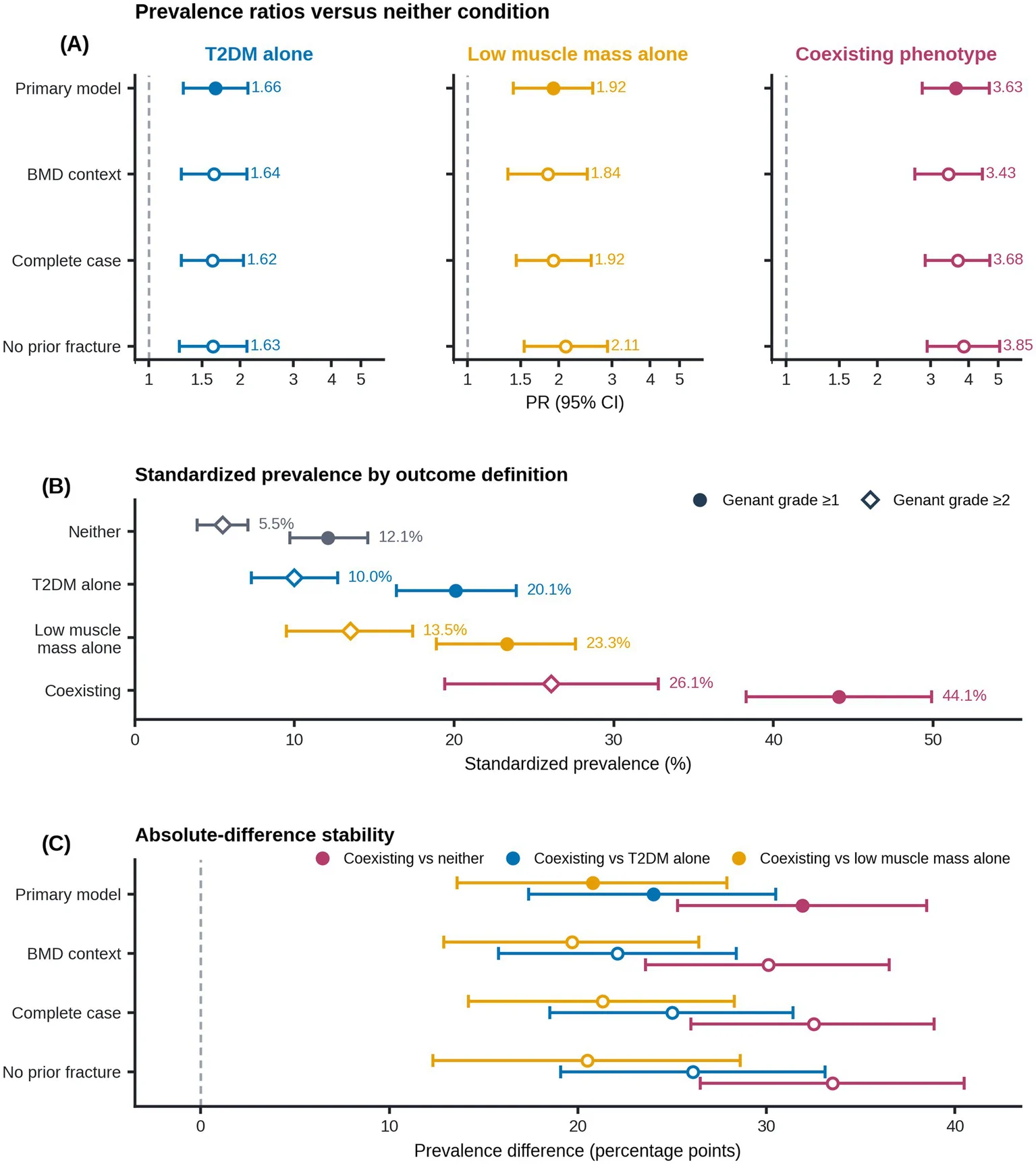
Stability of relative and absolute phenotype contrasts across retained analysis settings. **(A)** Prevalence ratios for T2DM alone, low muscle mass alone, and the coexisting phenotype versus neither condition in the primary, femoral-neck-BMD-context, complete-case, and observed-no-prior-fracture analyses. Filled points indicate the primary model and open points indicate the other retained analyses. The prevalence-ratio axes are shown on a common logarithmic scale. **(B)** Standardized prevalence of Genant grade ≥ 1 and Genant grade ≥ 2 radiographic vertebral fracture across the four phenotypes. In panel B, colors identify phenotypes and marker shape identifies outcome definition. **(C)** Prevalence differences for the coexisting phenotype versus neither condition, T2DM alone, and low muscle mass alone across the retained analysis settings. Filled points identify the primary model and open points identify the other retained analyses. No formal between-setting tests were performed. The BMD-context model is not a mediation analysis and does not establish independence from BMD. BMD, bone mineral density; CI, confidence interval; PR, prevalence ratio; T2DM, type 2 diabetes mellitus.

For Genant grade ≥ 2 radiographic vertebral fracture, PRs versus neither condition were 1.82 (95% CI, 1.29–2.58; *p* = 0.001), 2.45 (95% CI, 1.60–3.75; *p* < 0.001), and 4.75 (95% CI, 3.22–7.00; *p* < 0.001) for T2DM alone, low muscle mass alone, and the coexisting phenotype, respectively; these estimates are reported in Supplementary Table S5A. Standardized prevalence across the four phenotypes was 5.5, 10.0, 13.5, and 26.1% (Figure 3B; Supplementary Table S5A).

The complete-case analysis included 1,605 participants and 335 primary-outcome events. PRs versus neither condition were 1.62 (95% CI, 1.28–2.05), 1.92 (95% CI, 1.45–2.56), and 3.68 (95% CI, 2.88–4.70), respectively; all *p* < 0.001 (Figure 3A; Supplementary Table S5B). The observed-no-prior-fracture restriction included 1,480 participants and 303 events; the corresponding PRs were 1.63 (95% CI, 1.26–2.11), 2.11 (95% CI, 1.54–2.90), and 3.85 (95% CI, 2.92–5.07), respectively; all *p* < 0.001 (Figure 3A; Supplementary Table S5C).

The prevalence difference for the coexisting phenotype versus neither condition was 31.9 percentage points in the primary model, 30.1 in the BMD-context model, 32.5 in the complete-case analysis, and 33.5 in the observed-no-prior-fracture restriction. Across these settings, direct differences for the coexisting phenotype ranged from 22.1 to 26.1 percentage points versus T2DM alone and from 19.7 to 21.3 percentage points versus low muscle mass alone (Figure 3C; Supplementary Tables S4C, S5B,C).

## 4 Discussion

### 4.1 Principal findings

In this community-based cross-sectional analysis of 1,642 older adults, coexisting T2DM and DXA-defined low muscle mass marked a relatively small subgroup in which prevalent radiographic vertebral fracture events were concentrated. The coexisting phenotype comprised 14.1% of the analytic cohort but contained 31.5% of all Genant grade ≥ 1 events and 35.2% of Genant grade ≥ 2 events. It also had the highest observed and standardized prevalence. Compared with neither condition, the primary-model PR was 3.63 and the standardized prevalence difference was 31.9 percentage points.

Direct comparisons with the single-condition groups showed the same ordering. The coexisting phenotype had a primary-model PR of 2.19 versus T2DM alone and 1.89 versus low muscle mass alone, with direct prevalence differences of 24.0 and 20.8 percentage points. These estimates quantify cross-sectional differences between phenotype categories; they do not demonstrate biological synergy or that either condition caused the other.

Similar relative and absolute contrasts were observed when femoral-neck BMD was added as a contemporaneous context variable, when Genant grade ≥ 2 fracture was used as the outcome, in complete cases, and among participants observed without previous low-trauma fracture. This consistency supports the stability of the descriptive cross-sectional finding without extending it to temporal or causal inference.

### 4.2 Interpretation in relation to metabolic and muscle-health vulnerability

T2DM is associated with skeletal fragility that may not be captured by low areal BMD alone, and older adults with T2DM may have preserved or higher BMD despite greater fracture vulnerability (6–9, 16, 32–35). Low muscle mass, low strength, and impaired physical performance are distinct aspects of muscle-health decline and are linked in prior literature to falls, fractures, mobility limitation, and reduced functional reserve (10–12, 17–21, 23, 24, 36). The present findings are compatible with the clinical coexistence of these vulnerabilities (37), but the study did not test a biological mechanism or establish their temporal sequence.

The baseline profile helps contextualize the phenotype contrasts. Compared with the neither-condition group, participants in the coexisting group had lower BMD, SMI, handgrip strength, gait speed, 25(OH)D, and eGFR and higher HbA1c and bone-turnover-marker values. Because these measures were assessed concurrently, they provide descriptive context rather than evidence of mediation or a shared pathophysiologic pathway.

DXA-defined low muscle mass is not synonymous with clinical sarcopenia (22). Although all 472 participants with DXA-defined low muscle mass also met the full AWGS functional criterion in this dataset, that equivalence was specific to the analytic sample and does not change the exposure reconstructed from DXA. The full AWGS result is therefore reported as cross-validation, not as a second exposure model or a basis for interchangeable terminology.

### 4.3 Clinical and public-health interpretation

The contrast between cohort share and event share shows that prevalent radiographic vertebral fracture burden was not evenly distributed within this analytic sample. Because T2DM is routinely documented in community chronic-disease care and muscle health is increasingly considered in healthy-aging assessment, the coexisting phenotype may merit greater clinical and public-health attention as a marker of overlapping metabolic and muscle-health vulnerability. This perspective is consistent with person-centered aging frameworks that integrate metabolic and functional assessment rather than managing single conditions in isolation (38–40).

Clinical translation of this cross-sectional pattern requires prospective evaluation. Studies designed to assess diagnostic accuracy, screening yield, cost-effectiveness, and incident fracture outcomes are needed before the coexisting phenotype can inform vertebral-imaging strategies or individual-level decision rules.

### 4.4 Strengths and limitations

Strengths include the source-linked participant flow from community resident records, standardized DXA body-composition and BMD measurements, baseline vertebral imaging, and simultaneous reporting of cohort share, event share, observed prevalence, standardized prevalence, prevalence differences, and PRs. The analytic workflow incorporated 50 multiple-imputation datasets, community-cluster-robust covariance estimation, direct phenotype contrasts, and four complementary robustness analyses. Together, these features support transparent interpretation of both the relative and absolute cross-sectional phenotype contrasts.

First, the cross-sectional design links current phenotype status to prevalent rather than incident radiographic vertebral fracture. Temporal ordering among T2DM, low muscle mass, functional impairment, and vertebral fracture therefore remains unresolved, and the analysis does not estimate incident fracture. Genant grade 1 changes may include longstanding deformity or nonfracture morphologic variation; the grade ≥ 2 analysis provides a severity-oriented check but does not fully resolve this classification uncertainty (5). Vertebral images were assessed by trained readers with senior review of discrepant or uncertain cases, but inter-reader agreement was not quantified, so imaging reproducibility cannot be expressed numerically.

Second, participating centers and residents were enrolled through a structured nonprobability process, and analytic inclusion required ascertainment of both phenotype and imaging outcome. The phenotype distribution and prevalence estimates therefore characterize this analytic sample rather than the older population of Changchun. Selection at the site, participation, or ascertainment stages may have affected absolute prevalence and between-phenotype contrasts; its direction and magnitude are unknown.

Third, T2DM classification reflected previously diagnosed disease documented in community records and corroborated by reported physician diagnosis, with medication use as supporting information. HbA1c characterized contemporaneous glycemia, while cohort-wide biochemical screening was not used for classification. Previously unrecognized T2DM could therefore have been assigned to a non-T2DM phenotype, potentially attenuating or otherwise altering the phenotype contrasts. Longitudinal glycemic exposure and complete antidiabetic treatment trajectories were not characterized.

Fourth, the primary muscle exposure was DXA-defined low muscle mass rather than the full clinical sarcopenia construct. Although every participant with low muscle mass also met the full AWGS criterion in this sample, this sample-specific overlap should not be generalized. Calcium, phosphate, parathyroid hormone, alkaline phosphatase, and detailed histories of anti-osteoporosis treatment, calcium or vitamin D supplementation, and other bone-active therapies were not among the standardized variables used in this analysis. These factors may influence BMD, bone turnover, vitamin D status, and vertebral fracture prevalence and therefore remain potential sources of residual confounding (9, 41, 42).

Fifth, the primary adjustment set comprised age, sex, BMI, and current smoking, with femoral-neck BMD examined separately as contemporaneous skeletal context. Because CCI and mFI-5 were represented as totals and standard formulations may incorporate diabetes, they were omitted to avoid partial adjustment for T2DM. Residual confounding may remain from broader comorbidity, frailty, nutrition, inflammatory burden, lifetime mechanical loading, pain-related activity restriction, falls, and treatment. The BMD-context model is not a mediation analysis and should not be interpreted as demonstrating independence from BMD.

Finally, multiple imputation assumes that missingness is conditionally at random given the variables in the imputation model. Covariate missingness was low, and complete-case estimates were similar. External validity is most relevant to community-dwelling older adults recruited through comparable service-center networks and assessed with similar diabetes ascertainment, DXA, and vertebral imaging procedures. Replication in probability-based and prospective cohorts with broader metabolic and treatment phenotyping and quantified imaging reliability will clarify generalizability and temporal interpretation.

## 5 Conclusion

In this community-based sample, coexisting T2DM and DXA-defined low muscle mass delineated a phenotype in which prevalent radiographic vertebral fracture burden was concentrated beyond that observed with either condition alone. The principal contribution is therefore a phenotype-level view of fracture heterogeneity: metabolic disease and diminished muscle mass should be considered jointly when characterizing the distribution of existing vertebral fracture burden in older adults. Longitudinal and diagnostic studies are needed to determine whether this cross-sectional pattern has prognostic or clinical utility.

## Data Availability

The raw data supporting the conclusions of this article will be made available by the authors, without undue reservation.
