# Supplementary material for: Association of coexisting type 2 diabetes and DXA-defined low muscle mass with prevalent radiographic vertebral fracture in community-dwelling older adults: a cross-sectional study
Source: Front Public Health. 2026 Sep 16;14:1908757. doi: 10.3389/fpubh.2026.1908757 (PMC13623588; doi:10.3389/fpubh.2026.1908757)
Supplement: Supplementary file 1 [file Data_Sheet_1.docx]

Supplementary Material

# 1 Supplementary Figures and Tables

## Supplementary Figures

**Supplementary Figure S1. Participant identification, recruitment, baseline assessment, and analytic-cohort formation.**
The diagram shows study-setting and source-frame construction, the mutually exclusive first-exit counts at each recruitment and assessment stage, formation of the final analytic cohort, and classification of the cohort into four mutually exclusive T2DM-low-muscle-mass phenotypes. Resident records were assembled, merged, and deduplicated from multiple community sources within 16 participating community health service centers covering 36 administrative communities. Genant grade ≥1 VF counts denote participants with prevalent radiographic vertebral fracture. The aggregate analytic exclusion of 67 participants after completion of the site baseline assessment workflow was not subdivided because a source-supported reason-specific split was unavailable. Abbreviations: T2DM, type 2 diabetes mellitus; VF, vertebral fracture.

## Supplementary Tables

**Supplementary Table S1. Cross-validation of DXA-defined low muscle mass against the full AWGS 2019 classification.**

| **Classification/component** | **Rule** | ***n*** | **Role/interpretation** |
| --- | --- | --- | --- |
| DXA-defined low muscle mass | Men SMI <7.0 kg/m²; women SMI <5.4 kg/m²; strict < | 472 | Primary exposure component |
| Low handgrip strength | Men <28 kg; women <18 kg | 561 | Functional component |
| Slow gait | Gait speed <1.0 m/s | 954 | Functional component |
| Full AWGS 2019 classification | Low muscle mass AND (low grip OR slow gait) | 472 | Cross-validation only |
| Severe AWGS 2019 classification | Low muscle mass AND low grip AND slow gait | 365 | Descriptive only |
| Low muscle mass not meeting full AWGS | Deterministic discordance count | 0 | 0/472; 100.0% of low-muscle-mass participants met the full functional criterion in this dataset |

**Note:** The numerical equivalence of the reconstructed low-muscle-mass and full-AWGS classifications in this particular analytic sample is a data fact, not a reason to relabel the primary exposure. The primary exposure remains DXA-defined low muscle mass, and no duplicate association model was fitted using the full AWGS label. Abbreviations: AWGS, Asian Working Group for Sarcopenia; DXA, dual-energy X-ray absorptiometry; SMI, skeletal muscle index.

**Supplementary Table S2. Sources, assessment, operationalization, and final analytic role of retained variables.**

| **Variable** | **Role** | **Source/assessment** | **Operational definition or coding** | **Final analytic use** | **Transparency boundary** |
| --- | --- | --- | --- | --- | --- |
| T2DM | Primary exposure component | Community health-service records; survey interview corroboration by participant or family report when needed; glucose-lowering medication used as supporting information | Documented clinical diagnosis in community records or reported physician diagnosis; baseline HbA1c characterized glycemic status and was not used to re-diagnose T2DM | Four mutually exclusive phenotypes; not imputed | Fasting glucose and a complete objective reclassification algorithm were not recoverable; potential undiagnosed T2DM remains a limitation |
| DXA-defined low muscle mass | Primary exposure component | Hologic Discovery Wi whole-body DXA body-composition assessment; SMI = appendicular skeletal muscle mass/height² | Men SMI <7.0 kg/m²; women SMI <5.4 kg/m²; strict less-than rule | Four mutually exclusive phenotypes; not imputed | Primary exposure is low muscle mass, not a full sarcopenia diagnosis |
| Handgrip strength | Descriptive/full-AWGS cross-validation | Standardized baseline handgrip assessment | Low strength: men <28 kg; women <18 kg | Descriptive Table 1 and AWGS cross-validation only | Not entered in the primary association model |
| Gait speed | Descriptive/full-AWGS cross-validation | Standardized baseline gait-speed assessment | Slow gait: <1.0 m/s | Descriptive Table 1 and AWGS cross-validation only | Not entered in the primary association model |
| Full AWGS 2019 classification | Validation classification | Deterministic combination of source-derived low muscle mass, low handgrip strength, and slow gait | Low muscle mass AND (low grip OR slow gait); severe classification = all three present | Supplementary Table S1 cross-validation only | Does not replace or relabel the primary exposure and is not refitted as a duplicate association model |
| Prevalent radiographic vertebral fracture | Primary outcome | Baseline lateral thoracic and lumbar vertebral imaging; semiquantitative Genant assessment; uncertain readings resolved by senior review | At least one vertebra with highest Genant grade ≥1 | Primary outcome; not imputed | Does not establish osteoporotic or fragility etiology; reader-agreement statistics were unavailable |
| Genant grade ≥2 radiographic vertebral fracture | Key secondary outcome | Same baseline vertebral imaging assessment | At least one vertebra with highest Genant grade ≥2 | Key secondary-outcome model | Severity-oriented robustness; not an incident-fracture endpoint |
| Age | Primary covariate | Baseline assessment/recorded age in years | Continuous; 3-knot restricted cubic spline at 66.91, 73.00, and 81.49 years | Primary and all retained models | Retained continuously; no categorization was used |
| Sex | Primary covariate | Baseline record/interview | Binary female indicator | Primary and all retained models | Retained as a covariate in the corresponding models |
| BMI | Primary covariate | Measured body weight divided by measured height squared | Continuous; 3-knot restricted cubic spline at 20.8, 25.4, and 31.2 kg/m² | Primary and all retained models; missing values imputed | Retained continuously; no categorization was used |
| Current smoking | Primary covariate | Standardized baseline questionnaire binary field | Current smoking yes/no | Primary and all retained models; missing values imputed | Pack-years and exact historical smoking intensity were not reconstructed |
| Femoral-neck BMD | BMD-context covariate | Hologic Discovery Wi DXA hip scan | Continuous; 3-knot restricted cubic spline at 0.521, 0.683, and 0.835 g/cm² | Only in the separate BMD-context model; missing values imputed | Interpreted only as a contemporaneous BMD-context covariate |
| Previous low-trauma fracture | Descriptive/restriction variable | Standardized baseline questionnaire/recorded binary field | Observed yes/no; missing values remain ineligible for the observed-no-prior-fracture restriction | Table 1, MICE auxiliary information, and observed-no-prior-fracture restriction | Not a primary covariate; restriction eligibility was never imputed |
| Fall in previous year | Descriptive/MICE auxiliary | Standardized baseline questionnaire binary field | Recorded yes/no | Table 1 and MICE auxiliary information | Used only for descriptive and imputation-model support |
| Low physical activity | Descriptive/MICE auxiliary | Standardized baseline questionnaire-derived binary field | Binary field in the analysis dataset | Table 1 and MICE auxiliary information | Exact instrument wording/cutoff was not reconstructed and the variable is not used as a primary covariate |
| HbA1c | Descriptive/MICE auxiliary | Baseline laboratory result | Continuous percentage | Table 1 and MICE auxiliary information | Not used to define or reclassify T2DM |
| P1NP; beta-CTX; 25(OH)D; eGFR | Descriptive/MICE auxiliary | Baseline laboratory or derived final values | Continuous variables on reported units | Table 1 and MICE auxiliary information; not primary covariates | Used only as continuous descriptive or imputation-model support variables; calcium, phosphate, PTH, alkaline phosphatase, and detailed bone-active treatment data were unavailable |

**Abbreviations:** AWGS, Asian Working Group for Sarcopenia; BMD, bone mineral density; BMI, body mass index; beta-CTX, beta C-terminal telopeptide of type I collagen; DXA, dual-energy X-ray absorptiometry; eGFR, estimated glomerular filtration rate; HbA1c, glycated hemoglobin; MICE, multiple imputation by chained equations; P1NP, procollagen type I N-terminal propeptide; PTH, parathyroid hormone; SMI, skeletal muscle index; T2DM, type 2 diabetes mellitus; 25(OH)D, 25-hydroxyvitamin D.

**Supplementary Table S3. Variable-specific availability and missingness in the analytic cohort.**

| **Variable** | **Available, *n*** | **Missing, *n*** | **Missing, %** |
| --- | --- | --- | --- |
| Age, years | 1642 | 0 | 0.00 |
| Sex | 1642 | 0 | 0.00 |
| T2DM status | 1642 | 0 | 0.00 |
| SMI, kg/m² | 1642 | 0 | 0.00 |
| DXA-defined low muscle mass | 1642 | 0 | 0.00 |
| Baseline prevalent radiographic vertebral fracture | 1642 | 0 | 0.00 |
| BMI, kg/m² | 1618 | 24 | 1.46 |
| Current smoking | 1629 | 13 | 0.79 |
| Previous low-trauma fracture | 1626 | 16 | 0.97 |
| Fall in previous year | 1620 | 22 | 1.34 |
| Low physical activity | 1611 | 31 | 1.89 |
| Femoral-neck BMD, g/cm² | 1602 | 40 | 2.44 |
| Lumbar-spine BMD, g/cm² | 1603 | 39 | 2.38 |
| Handgrip strength, kg | 1642 | 0 | 0.00 |
| Gait speed, m/s | 1642 | 0 | 0.00 |
| HbA1c, % | 1642 | 0 | 0.00 |
| P1NP, ng/mL | 1591 | 51 | 3.11 |
| beta-CTX, ng/mL | 1593 | 49 | 2.98 |
| 25(OH)D, ng/mL | 1596 | 46 | 2.80 |
| eGFR, mL/min/1.73 m² | 1621 | 21 | 1.28 |

**Note:** The denominator is *N*=1,642. T2DM duration is not shown as a missingness variable because it is structurally not applicable to participants without T2DM; it is summarized only among participants with T2DM in Table 1. Missing covariate values were addressed using 50 completed datasets from multiple imputation by chained equations. The exposure, outcomes, and restriction eligibility were not imputed.

**Supplementary Table S4. Unadjusted, primary, and femoral-neck-BMD-context results.**

**S4A. Prevalence ratios**

| **Analysis setting** | **Contrast** | ***N*** | **Events** | **PR (95% CI)** | ***P*** |
| --- | --- | --- | --- | --- | --- |
| Unadjusted descriptive model | T2DM alone vs neither condition | 1642 | 343 | 1.79 (1.41–2.26) | <0.001 |
| Unadjusted descriptive model | Low muscle mass alone vs neither condition | 1642 | 343 | 2.09 (1.56–2.81) | <0.001 |
| Unadjusted descriptive model | Coexisting phenotype vs neither condition | 1642 | 343 | 4.03 (3.11–5.22) | <0.001 |
| Unadjusted descriptive model | Coexisting phenotype vs T2DM alone | 1642 | 343 | 2.25 (1.81–2.80) | <0.001 |
| Unadjusted descriptive model | Coexisting phenotype vs low muscle mass alone | 1642 | 343 | 1.93 (1.52–2.44) | <0.001 |
| Primary multiple-imputation model | T2DM alone vs neither condition | 1642 | 343 | 1.66 (1.30–2.12) | <0.001 |
| Primary multiple-imputation model | Low muscle mass alone vs neither condition | 1642 | 343 | 1.92 (1.42–2.59) | <0.001 |
| Primary multiple-imputation model | Coexisting phenotype vs neither condition | 1642 | 343 | 3.63 (2.81–4.68) | <0.001 |
| Primary multiple-imputation model | Coexisting phenotype vs T2DM alone | 1642 | 343 | 2.19 (1.76–2.72) | <0.001 |
| Primary multiple-imputation model | Coexisting phenotype vs low muscle mass alone | 1642 | 343 | 1.89 (1.51–2.37) | <0.001 |
| Femoral-neck-BMD-context model | T2DM alone vs neither condition | 1642 | 343 | 1.64 (1.28–2.11) | <0.001 |
| Femoral-neck-BMD-context model | Low muscle mass alone vs neither condition | 1642 | 343 | 1.84 (1.36–2.49) | <0.001 |
| Femoral-neck-BMD-context model | Coexisting phenotype vs neither condition | 1642 | 343 | 3.43 (2.66–4.44) | <0.001 |
| Femoral-neck-BMD-context model | Coexisting phenotype vs T2DM alone | 1642 | 343 | 2.09 (1.69–2.58) | <0.001 |
| Femoral-neck-BMD-context model | Coexisting phenotype vs low muscle mass alone | 1642 | 343 | 1.86 (1.50–2.32) | <0.001 |

**S4B. Standardized prevalence**

| **Analysis setting** | **Phenotype** | ***N*** | **Events** | **Standardized prevalence (95% CI)** | ***P*** |
| --- | --- | --- | --- | --- | --- |
| Primary multiple-imputation model | Neither condition | 1642 | 343 | 12.1% (9.7%–14.6%) | — |
| Primary multiple-imputation model | T2DM alone | 1642 | 343 | 20.1% (16.4%–23.9%) | — |
| Primary multiple-imputation model | Low muscle mass alone | 1642 | 343 | 23.3% (18.9%–27.6%) | — |
| Primary multiple-imputation model | Coexisting phenotype | 1642 | 343 | 44.1% (38.3%–49.9%) | — |
| Femoral-neck-BMD-context model | Neither condition | 1642 | 343 | 12.3% (9.9%–14.8%) | — |
| Femoral-neck-BMD-context model | T2DM alone | 1642 | 343 | 20.3% (16.5%–24.1%) | — |
| Femoral-neck-BMD-context model | Low muscle mass alone | 1642 | 343 | 22.7% (18.5%–27.0%) | — |
| Femoral-neck-BMD-context model | Coexisting phenotype | 1642 | 343 | 42.4% (36.8%–48.0%) | — |

**S4C. Prevalence differences**

| **Analysis setting** | **Contrast** | ***N*** | **Events** | **Prevalence difference (95% CI)** | ***P*** |
| --- | --- | --- | --- | --- | --- |
| Primary multiple-imputation model | T2DM alone vs neither condition | 1642 | 343 | 8.0 (3.9–12.0) percentage points | <0.001 |
| Primary multiple-imputation model | Low muscle mass alone vs neither condition | 1642 | 343 | 11.1 (5.7–16.5) percentage points | <0.001 |
| Primary multiple-imputation model | Coexisting phenotype vs neither condition | 1642 | 343 | 31.9 (25.3–38.5) percentage points | <0.001 |
| Primary multiple-imputation model | Coexisting phenotype vs T2DM alone | 1642 | 343 | 24.0 (17.4–30.5) percentage points | <0.001 |
| Primary multiple-imputation model | Coexisting phenotype vs low muscle mass alone | 1642 | 343 | 20.8 (13.6–27.9) percentage points | <0.001 |
| Femoral-neck-BMD-context model | T2DM alone vs neither condition | 1642 | 343 | 7.9 (3.8–12.1) percentage points | <0.001 |
| Femoral-neck-BMD-context model | Low muscle mass alone vs neither condition | 1642 | 343 | 10.4 (5.1–15.7) percentage points | <0.001 |
| Femoral-neck-BMD-context model | Coexisting phenotype vs neither condition | 1642 | 343 | 30.1 (23.6–36.5) percentage points | <0.001 |
| Femoral-neck-BMD-context model | Coexisting phenotype vs T2DM alone | 1642 | 343 | 22.1 (15.8–28.4) percentage points | <0.001 |
| Femoral-neck-BMD-context model | Coexisting phenotype vs low muscle mass alone | 1642 | 343 | 19.7 (12.9–26.4) percentage points | <0.001 |

**Note:** The unadjusted model is descriptive. The primary model includes age and BMI as three-knot restricted cubic splines, sex, and current smoking. The BMD-context model adds femoral-neck BMD as a three-knot restricted cubic spline. Both multiple-imputation models use 50 completed datasets and 36-community cluster-robust covariance with the CR1 small-sample correction. Direct coexisting-versus-single-condition comparisons are contrasts between levels of the four-category exposure and do not imply biological synergy. Prevalence differences were calculated directly from unrounded pooled marginal estimates; displayed rounded standardized prevalences need not subtract exactly to the displayed difference.

**Supplementary Table S5. Key secondary-outcome and sensitivity results.**

**S5A. Genant grade ≥2 key secondary outcome**

| **Estimate type** | **Contrast/group** | ***N*** | **Events** | **Estimate (95% CI)** | ***P*** |
| --- | --- | --- | --- | --- | --- |
| PR | T2DM alone vs neither condition | 1642 | 182 | 1.82 (1.29–2.58) | 0.001 |
| PR | Low muscle mass alone vs neither condition | 1642 | 182 | 2.45 (1.60–3.75) | <0.001 |
| PR | Coexisting phenotype vs neither condition | 1642 | 182 | 4.75 (3.22–7.00) | <0.001 |
| PR | Coexisting phenotype vs T2DM alone | 1642 | 182 | 2.60 (1.72–3.93) | <0.001 |
| PR | Coexisting phenotype vs low muscle mass alone | 1642 | 182 | 1.93 (1.29–2.90) | 0.002 |
| Standardized prevalence | Neither condition | 1642 | 182 | 5.5% (3.9%–7.1%) | — |
| Standardized prevalence | T2DM alone | 1642 | 182 | 10.0% (7.3%–12.7%) | — |
| Standardized prevalence | Low muscle mass alone | 1642 | 182 | 13.5% (9.5%–17.4%) | — |
| Standardized prevalence | Coexisting phenotype | 1642 | 182 | 26.1% (19.4%–32.8%) | — |
| Prevalence difference | T2DM alone vs neither condition | 1642 | 182 | 4.5 (1.8–7.3) percentage points | 0.002 |
| Prevalence difference | Low muscle mass alone vs neither condition | 1642 | 182 | 8.0 (3.7–12.3) percentage points | <0.001 |
| Prevalence difference | Coexisting phenotype vs neither condition | 1642 | 182 | 20.6 (13.7–27.5) percentage points | <0.001 |
| Prevalence difference | Coexisting phenotype vs T2DM alone | 1642 | 182 | 16.1 (8.3–23.8) percentage points | <0.001 |
| Prevalence difference | Coexisting phenotype vs low muscle mass alone | 1642 | 182 | 12.6 (4.5–20.7) percentage points | 0.003 |

**S5B. Complete-case sensitivity analysis**

| **Estimate type** | **Contrast/group** | ***N*** | **Events** | **Estimate (95% CI)** | ***P*** |
| --- | --- | --- | --- | --- | --- |
| PR | T2DM alone vs neither condition | 1605 | 335 | 1.62 (1.28–2.05) | <0.001 |
| PR | Low muscle mass alone vs neither condition | 1605 | 335 | 1.92 (1.45–2.56) | <0.001 |
| PR | Coexisting phenotype vs neither condition | 1605 | 335 | 3.68 (2.88–4.70) | <0.001 |
| PR | Coexisting phenotype vs T2DM alone | 1605 | 335 | 2.27 (1.84–2.81) | <0.001 |
| PR | Coexisting phenotype vs low muscle mass alone | 1605 | 335 | 1.91 (1.53–2.38) | <0.001 |
| Standardized prevalence | Neither condition | 1605 | 335 | 12.1% (9.8%–14.5%) | — |
| Standardized prevalence | T2DM alone | 1605 | 335 | 19.6% (16.1%–23.1%) | — |
| Standardized prevalence | Low muscle mass alone | 1605 | 335 | 23.3% (19.0%–27.7%) | — |
| Standardized prevalence | Coexisting phenotype | 1605 | 335 | 44.6% (38.9%–50.3%) | — |
| Prevalence difference | T2DM alone vs neither condition | 1605 | 335 | 7.5 (3.7–11.3) percentage points | <0.001 |
| Prevalence difference | Low muscle mass alone vs neither condition | 1605 | 335 | 11.2 (6.0–16.4) percentage points | <0.001 |
| Prevalence difference | Coexisting phenotype vs neither condition | 1605 | 335 | 32.5 (26.0–38.9) percentage points | <0.001 |
| Prevalence difference | Coexisting phenotype vs T2DM alone | 1605 | 335 | 25.0 (18.5–31.4) percentage points | <0.001 |
| Prevalence difference | Coexisting phenotype vs low muscle mass alone | 1605 | 335 | 21.3 (14.2–28.3) percentage points | <0.001 |

**S5C. Observed-no-prior-low-trauma-fracture restriction**

| **Estimate type** | **Contrast/group** | ***N*** | **Events** | **Estimate (95% CI)** | ***P*** |
| --- | --- | --- | --- | --- | --- |
| PR | T2DM alone vs neither condition | 1480 | 303 | 1.63 (1.26–2.11) | <0.001 |
| PR | Low muscle mass alone vs neither condition | 1480 | 303 | 2.11 (1.54–2.90) | <0.001 |
| PR | Coexisting phenotype vs neither condition | 1480 | 303 | 3.85 (2.92–5.07) | <0.001 |
| PR | Coexisting phenotype vs T2DM alone | 1480 | 303 | 2.37 (1.86–3.01) | <0.001 |
| PR | Coexisting phenotype vs low muscle mass alone | 1480 | 303 | 1.82 (1.41–2.36) | <0.001 |
| Standardized prevalence | Neither condition | 1480 | 303 | 11.8% (9.3%–14.2%) | — |
| Standardized prevalence | T2DM alone | 1480 | 303 | 19.1% (15.1%–23.2%) | — |
| Standardized prevalence | Low muscle mass alone | 1480 | 303 | 24.8% (19.5%–30.1%) | — |
| Standardized prevalence | Coexisting phenotype | 1480 | 303 | 45.3% (39.3%–51.2%) | — |
| Prevalence difference | T2DM alone vs neither condition | 1480 | 303 | 7.4 (3.2–11.5) percentage points | 0.001 |
| Prevalence difference | Low muscle mass alone vs neither condition | 1480 | 303 | 13.0 (6.9–19.2) percentage points | <0.001 |
| Prevalence difference | Coexisting phenotype vs neither condition | 1480 | 303 | 33.5 (26.5–40.5) percentage points | <0.001 |
| Prevalence difference | Coexisting phenotype vs T2DM alone | 1480 | 303 | 26.1 (19.1–33.1) percentage points | <0.001 |
| Prevalence difference | Coexisting phenotype vs low muscle mass alone | 1480 | 303 | 20.5 (12.3–28.6) percentage points | <0.001 |

**Note:** The complete-case sensitivity analysis includes *N*=1,605 and 335 events. The observed-no-prior-fracture restriction includes *N*=1,480 and 303 events; 16 participants with missing prior-fracture status were not treated as having no prior fracture, and restriction eligibility was not imputed. The Genant grade ≥2 analysis includes *N*=1,642 and 182 events. All settings retain the same four-category exposure and the primary-model covariate structure, except that the complete-case analysis does not use imputation. Prevalence differences were calculated from unrounded pooled marginal estimates.
